# The Development and Testing of a Chemotherapy-Induced Phlebitis Severity (CIPS) Scale for Patients Receiving Anthracycline Chemotherapy for Breast Cancer

**DOI:** 10.3390/jcm9030701

**Published:** 2020-03-05

**Authors:** Valerie Harris, Meinir Hughes, Rosie Roberts, Gina Dolan, E. Mark Williams

**Affiliations:** 1 Velindre Cancer Centre, Velindre Road, Cardiff CF14 2TL, UK; Meinir.Hughes2@wales.nhs.uk (M.H.); Rosie.roberts3@wales.nhs.uk (R.R.); 2 Faculty of Life Sciences and Education, University of South Wales, Pontypridd CF37 1DL, UK; gina.dolan@southwales.ac.uk (G.D.); mark.williams@southwales.ac.uk (E.M.W.)

**Keywords:** chemical phlebitis, anthracycline, breast cancer, phlebitis scale, chemotherapy

## Abstract

A chemotherapy induced phlebitis severity (CIPS) scale was developed in patients receiving anthracycline chemotherapy for breast cancer. A five-point severity scoring scale for chemotherapy-induced phlebitis was tested for inter-rater reliability. Ease of use was observed through timing assessments and a review of the completeness of documentation. A comparison of CIPS scale grade with participant reported severity scores was made. The final version was tested for inter-rater reliability, with 122 patient assessments. There was an 89.3% (109 of 122) agreement between the assessors (κ = 0.82, SE ± 0.042, 95% CI 0.74–0.90). Mean time to complete the scale was 1 min 36 s and documentation was fully completed for 98% of assessments. Patient reported severity closely matched the CIPS grade (κ = 0.54, SE ± 0.045, 95% CI 0.46–0.63). This new scale provides a list of symptoms associated with chemotherapy phlebitis, which can be scored quickly and accurately. It provides a reliable method for assessing chemotherapy-induced phlebitis, enabling a better understanding of its impact on patients’ quality of life, and to inform the appropriate choice of peripheral or central intravenous administration. Multicentre testing of the CIPS scale is recommended.

## 1. Introduction

Despite the use of new targeted therapies and improved cancer detection, chemotherapy treatment continues to play a major role in cancer treatment [1]. It is well documented that the administration of chemotherapy can result in significant toxicities and complications. One recognised consequence of peripheral administration of some intravenous chemotherapy is venous irritation leading to phlebitis and thrombophlebitis resulting from the chemical effects of the drug on the vein wall [2,3]. Chemical phlebitis causes symptoms such as pain, swelling, redness and, at worst, hardening and sclerosis of the vein resulting in a palpable venous cord and thrombosis of the upper extremity veins [4,5]. It has been recommended that a central venous catheter (CVC) should be used for the administration of drugs which are known to cause significant venous irritation and phlebitis [6]. However, the decision to place a CVC needs to be carefully assessed, taking into consideration the risk of infection and thrombosis associated with central line placement [7], compared with the severity and impact of phlebitis.

At their most severe, phlebitis symptoms can last for months following the completion of chemotherapy, which can have a considerable impact on patients’ quality of life and their ability to carry out normal daily activities [8]. However, for patients who only experience mild and transient symptoms, these may be viewed as an inconvenience requiring no clinical intervention, which would not justify the additional risks and costs of placement of a central venous catheter [9]. The reported rates of chemotherapy-induced phlebitis vary considerably from 3% to 89%, with the exact incidence and severity being difficult to assess due to the different methodology and assessment scales used [10,11]. When trying to identify a suitable scale to assess the severity of chemotherapy-induced phlebitis, it became evident that, despite the existence of a variety of scoring and assessment tools, there is, as yet, no existing phlebitis assessment scale which specifically and comprehensively measures the effect of chemical phlebitis as a result of peripheral chemotherapy infusions [12].

Commonly used phlebitis assessment scales, such as the visual infusion phlebitis (VIP) scale [13], the phlebitis scale [14], and the Baxter scale [15], are designed to assess symptoms of phlebitis from an indwelling peripheral cannula to identify when it should be replaced, and not to assess the delayed effects of chemical phlebitis following chemotherapy. Rittenberg et al. (1995) developed the only available chemotherapy specific tool used for grading the venous irritation associated with vinorelbine chemotherapy [16]. However, this scale does not include some of the common symptoms of chemical phlebitis, such as a palpable venous cord, or use a scale to clarify levels of pain which may increase the risk of subjectivity in assessment. The National Cancer Institute (NCI) Common Terminology Criteria for Adverse Events (CTCAE, version 4.03), which is widely used to grade chemotherapy toxicities, simply grades phlebitis as grade 2 if present, without providing any criteria for severity of symptoms.

The validity and reliability of all available phlebitis assessment scales has been questioned by Ray-Barruel et al. (2015) and Göransson et al. (2017), who both conclude that no phlebitis scale has been thoroughly validated, and raise concerns about whether they are fit for purpose [17,18]. Marsh et al. (2015) assessed inter-rater agreements between nurses using ten different phlebitis scales with 210 patients [12]. They established that inter-rater agreement across the phlebitis scales was generally poor, the Rittenberg scale was identified as one of the best with >80% agreement, and they recommended further development of this tool.

This study aims to develop a chemotherapy induced phlebitis severity (CIPS) scale, which provides a comprehensive and reliable measure of the severity of chemotherapy-induced phlebitis, which can be used to help assess the impact on patients and inform the choice of intravenous access.

## 2. Methods

The CIPS scale was developed by a team of three specialist nurses with expertise in breast cancer, intravenous access and chemotherapy, all with over 20 years’ clinical experience in oncology and qualified at Master’s level. An expert nurse is deemed to possess widespread clinical experience and an extensive body of knowledge [19].

The CIPS scale was designed with five levels of symptom grades, in order of severity from zero to four. The choice of grading levels was based on the grade descriptors used by the National Cancer Institute Common Terminology Criteria for Adverse Events version 4 (NCI-CTCAE, 2009), which is well known to clinical staff working in oncology [20]. Grade zero denotes no symptoms; grade one mild, grade two moderate, grade three marked and grade four severe. In addition, the effect on arm function, which is a key distinguishing feature of Rittenberg’s (1995) scale, was considered important for the CIPS scale to ensure that the impact of chemotherapy-induced phlebitis on patients was taken into consideration when grading the severity of symptoms [16]. The final wording chosen to describe the effect on function in the CIPS scale was influenced by the NCI-CTCAE, which uses the effect of symptoms on activities of daily living (ADL) as a key measure to indicate the severity and impact of symptoms on patients [20].

**Figure 1:**
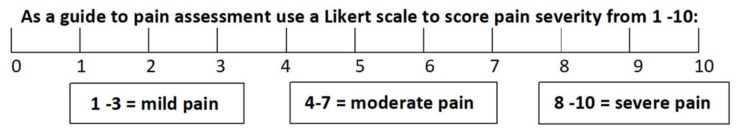
Likert scale for pain assessment.

The scale includes the widely recognised symptoms of phlebitis redness, swelling and pain [17]. The CIPS scale also includes the addition of other symptoms, not consistently found in generic phlebitis assessments tools, but frequently seen with chemotherapy-induced chemical phlebitis, namely; tethering, palpable venous cord, vein discolouration and impaired arm function. To better evaluate the pain experienced by patients, a Likert scale was used to determine the severity of the discomfort from 0–10, with 10 being the most severe (Figure 1). A score of 1–3 was described as mild pain, 4–7 as moderate and 8–10 as severe. This pain score was then utilised within the assessment scale to assist in the determination of the severity of the overall symptoms.

The CIPS scale was initially piloted in 2015 in a prospective audit of 51 patients receiving 75 cycles of chemotherapy to identify the severity of the symptoms of chemical phlebitis. During this audit, the scale was reviewed and amended from version 1–3 to improve the definitions of the symptoms of chemical phlebitis within each grade. The scale was then tested for inter-rater reliability by the three expert nurses for a further 85 patient assessments. Inter-rater reliability is used to establish whether a scale or measure produces consistent results when used by different assessors and is recommended as one of the key methods to test the reliability of phlebitis assessment scales [12,17].

**Figure 2:**
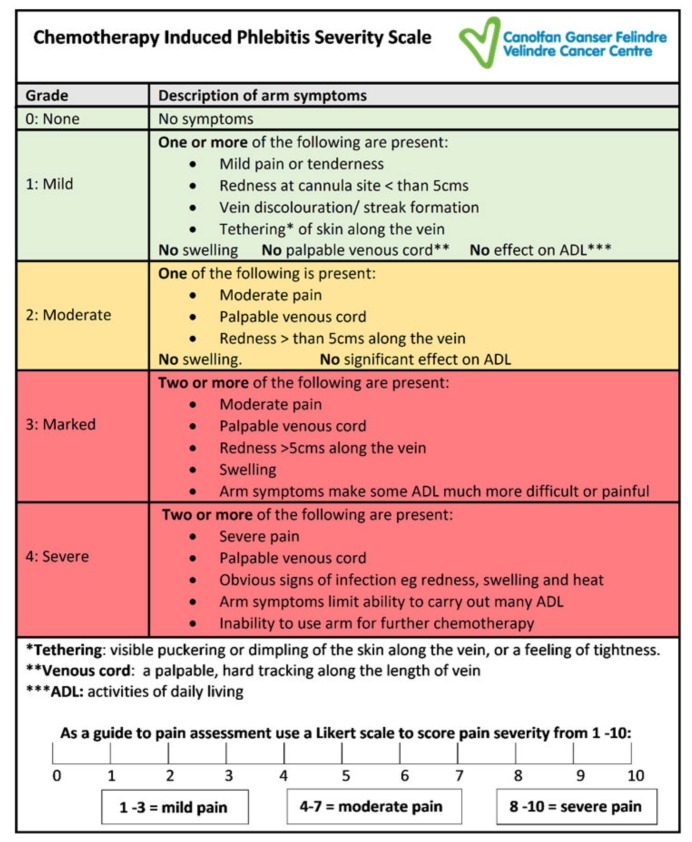
The chemotherapy-induced phlebitis severity scale.

During this testing, a series of minor amendments were made to the wording used, clarifying the grade descriptors to improve consistency in interpretation of symptoms. For example, replacing ‘impairment of arm function or movement’ with ‘effect on activities of daily living (ADL)’, to be consistent with the NCI CTCAE terminology, and the inclusion of ‘inability to use arm for further chemotherapy’ in grade 4, to reflect Rittenberg’s grading scale [20]. The final version of the phlebitis scale (Figure 2) was used for testing for inter-rater reliability in this study. Ethical approval to use the CIPS scale was included as part of a study on anthracycline induced phlebitis (REC reference: 16/WA/0074) [9].

Six practitioners were recruited to take part in the inter-rater reliability assessments of the final version of the scale. All were nurses practising in breast cancer, chemotherapy or intravenous access. They received guidance on the use of the CIPS scale from the initial group of expert nurses who developed the scale. Following a verbal agreement from the patient, two assessors used the measurement scale to grade phlebitis in separate assessments a few minutes apart.

In addition to inter-rater reliability assessments, feasibility of use in practice was tested by timing the assessment using the scale and reviewing whether all documentation on the assessment form was complete. The CIPS scale grade recorded by clinical staff was then compared with participant reported severity of symptoms.

A statistical analysis was performed using the Statistical Package for Social Science software (V25, SPSS, IBM, New York, NY, USA).

## 3. Results

A total of 122 assessments were performed during the inter-rater reliability testing process from November 2016–March 2018. A convenience sample of patients, attending for routine assessment between anthracycline chemotherapy treatments, were used for the testing.

The inter-rater agreement for all assessors, with grade given on review, was high at 86.9% (106 out of 122), Cohen’s kappa (κ), 0.82 (SE 0.04, 95% CI’s 0.74–0.90) (Table 1).

The time taken to complete the assessment for a nurse experienced with using the scale was measured for 25 assessments (Table 2). The mean time to complete was 1 min 36 s (range 42 s–2 min 51 s). Out of 122 assessments, assessor 1 fully completed 98% (120 of 122) of the assessment forms and assessor 2 fully completed 97% (118 of 122) of the assessment forms.

After development and testing for inter-rater reliability the final version of CIPS was used for a study of anthracycline induced phlebitis [9]. Included in this study was a questionnaire, enabling patients to self- report and score any phlebitis symptoms, which was used in parallel with the staff assessment of symptoms using the CIPS scale. The patient reported severity scores compared to the staff CIPS scale grading (Table 3) demonstrated a good level of agreement (κ = 0.54, SE ± 0.045, 95% CI 0.46–0.63). As the patient reported severity score increased, the severity of the CIPS scale grade increased by 77% (57 of 66), and participants reporting no symptoms were graded as CIPS scale 0 or 1, and all 7 patients who reported severe symptoms were graded by staff as CIPS grade 3 or 4.

## 4. Discussion

The choice of the intravenous access route to administer chemotherapy should be made after an informed discussion with the patient about the risks and benefits of both peripheral and central venous administration. The potential risks of infection and thrombosis with central venous catheters are well documented [7,21], but it is currently difficult to provide patients with an accurate understanding of the potential severity and impact of chemotherapy-related phlebitis, which may influence venous access choices. Despite the number of assessment scales available to monitor phlebitis, there is no suitable scale that can be used specifically to assess and provide accurate information about the frequency and severity of chemotherapy-related chemical phlebitis. A standardised tool available for assessing chemotherapy-induced phlebitis as opposed to infective phlebitis would allow the routine reporting of this condition and provide prevalence rates to be collected at a local and national level; something which is not currently possible.

From a cancer perspective, the National Cancer Institute’s (NCI) Common Terminology Criteria for Adverse Events (CTCAE), which is extensively used to standardise the assessment of chemotherapy-related toxicities, fails to adequately address the issues of post treatment phlebitis [20]. This was also recognised by Rittenberg et al. (1995) when studying venous irritation resulting from peripheral administration of the chemotherapy drug vinorelbine [16]. As the NCI CTCAE phlebitis grading did not provide any opportunity to grade the severity of symptoms, Rittenberg et al. developed the Venous Irritation Record (VIR). The VIR devised specifically for chemotherapy-related phlebitis described common symptoms of phlebitis such as redness, swelling and pain but importantly also included impairment of arm function to enable a better assessment of the impact on the patient. However, the assessment of pain in the VIR is based on the level of analgesia required, which is not a widely recognised method of assessing pain, and additional symptoms such as venous cord formation, tethering and streak formation that can determine the presence and severity of chemical phlebitis were not included. The CIPS scale has incorporated these symptoms along with a Likert scale to measure the level of discomfort and pain experienced (Figure 1). This allows a more comprehensive approach to the assessment of chemotherapy-induced phlebitis

When introducing any new assessment scale into clinical practice, it is important to consider how feasible it is for busy clinical staff to use. If an assessment scale is not quick and easy to use, staff compliance with undertaking accurate assessments will be poor, and this will adversely affect its reliability in practice. The significance of assessing for feasibility was acknowledged by Ray-Barruel et al. (2014) in their systematic review of phlebitis assessment measures, recommending that factors such as the length of time to complete, as the ease of use and clarity of instructions were key indicators of excellence in clinical measurement scales [17]. The feasibility of the CIPS scale was primarily evaluated by recording the time from start to completion of assessment. The average time was 1 min 30 s, with the shorter assessment times being predominately associated with the higher or lower grades. Grade two, on average, took longer (2 min 10 s), and this may be explained by the additional time taken to evaluate the significance of symptoms, which clearly did not meet the obvious criteria for the lowest or highest grades. Interestingly, Grade 2 also had the greatest inconsistencies of assessment, possibly because it was the midpoint of the scale, and symptoms may be a combination of lower and higher individually graded symptoms. Feasibility and ease of use in practice were also demonstrated by the high number of assessment forms with documentation fully completed, which was between 97%–98%.

Reliability of the CIPS scale was tested using inter-rater agreement, which was strong with the items tested, demonstrating a high degree of agreement between assessors (Table 1). Overall, there was 89.3% (109 of 122) agreement between the assessors (κ = 0.82, SE ± 0.042, 95% CI 0.74–0.90). Although the number of assessments recording grade 3 or 4 was small (16 of 122), there was 100% agreement between assessors, giving some confidence that the scale enables staff to consistently identify the most severe symptoms. There were some inconsistencies in the assessment of a small number of patients, and as with most assessment tools, there will inevitably be some degree of inconsistency, as it relies on individual clinical judgment that can lead to subjectivity. Steps were taken to attempt to eliminate this, such as the use of a Likert scale to measure pain, and the inclusion of definitions of ‘tethering’ and ‘palpable venous cord’ to guide the users’ assessment. In addition, the immediacy of the two assessments reduces the potential for conflicting findings, as a result of a time-lapse.

There was a variation between the levels of pain recorded between the first and second assessor in four cases. An increase in the level of pain on the second assessment may be a result of the patient becoming more aware of the symptoms, or that the physical palpation by the first assessor increased the levels of pain, and the possibility of this was also identified by Marsh et al. (2015) [12] However, the reduction in the level of pain from assessor 1 to assessor 2 was unexpected, but could be explained by the indecisiveness of the patient being assessed, as the level of pain bordered between points on the Likert pain score. The need of training to use the CIPS scale was highlighted by the small number of occasions, when one of the assessors failed to recognise tethering or the presence of a palpable venous cord. The failure to identify painless tethering led us to recognise the importance of examining the arm when fully extended, to facilitate the identification of tethering.

A key priority during the development of the CIPS scale was to include measurement of the impact of chemical phlebitis on the patient. The effects on activities of daily living which reflect quality of life are a key feature of the NCI’s model of toxicity assessment, which is used extensively in cancer care. However very few of the current phlebitis measuring tools include this issue, with the exception of the tool developed by Rittenberg et al. (1995), who included a question concerning arm function. The CIPS scale extends this to measuring whether the limitations to daily activities are moderate or significant, along with the level of pain experienced, which can help demonstrate the negative impact chemical phlebitis has on quality of life and the ability to perform every day functions [16]. It also relies on the patient to report the impact on activities of daily living (ADL) with patient reported measure being increasingly recognised as essential to evaluation of symptoms experienced [22].

The comparison of patient reported severity of symptoms with staff assessment of grade using the CIPS scale (Table 3) demonstrated a good level of agreement (κ = 0.54, SE ± 0.045, 95% CI 0.46–0.63). A small number of patients who reported no symptoms were graded by staff as having grade 2 or 3 symptoms—this appears to be related to patients who either underreported symptoms; for example, not identifying discomfort as pain, or failing to identify hardness of the vein (palpable venous cord). However, all patients reporting severe symptoms were given a CIPS grade of either 3 or 4, and 95% (52 of 55) of those reporting moderate symptoms were given CIPS grades of 2 or above. This can be seen as providing a degree of face validity, as defined by Mokkink et al. (2012), in the Consensus based Standards for the selection of health Measurement Instruments manual (COSMIN), by confirming the ability of the scale to closely reflect the reality of the patients’ experience of chemotherapy-induced phlebitis [23].

### Limitations

Primarily only patients with breast cancer patients receiving anthracycline chemotherapy were chosen to test the CIPS scale. Further evaluation with other chemotherapy regimens and other larger patient groups would be recommended to assess its applicability to all chemotherapy agents and to ensure it is a reliable effective measurement of chemotherapy-induced phlebitis. It is recognised that the individuals involved in completing the CIPS scale were experienced chemotherapy or breast cancer specialist practitioners with a thorough understanding of the signs of phlebitis and how to clinically diagnose its symptoms. Further information concerning the feasibility of the CIPS scale could be drawn from the evaluation of nurses with varied levels of experience completing the tool. To further establish the validity of the CIPS scale, it should be tested in parallel with the Rittenberg scale [16], the only other published chemotherapy specific phlebitis scale. It is recommended that the inter-rater reliability, feasibility of use in practice, and ability to closely reflect patient reported symptoms of both scales should be compared.

## 5. Conclusions

The aim of this study was to test a newly devised scale designed specifically to identify and measure the severity of chemotherapy-related chemical phlebitis. The CIPS scale includes a comprehensive list of recognised symptoms to grade the severity of phlebitis and the effects on the patients’ activities of daily living. The CIPS scale is easy, quick, reliable and robust to use in the clinic and has been shown to closely reflect the breast cancer patient’s experience of chemotherapy- induced phlebitis.

Although the focus throughout the testing was on phlebitis-related to anthracycline chemotherapy, the CIPS scale could potentially be used in the assessment of all chemotherapy agents. It is recommended that for a more complete patient assessment chemical phlebitis should be recorded routinely along with other chemotherapy-related toxicities. This will enable a greater understanding of the impact of chemotherapy treatment and provide data about phlebitis rates and severity to inform evidence-based discussions with patients about the choice of central or peripheral venous access for chemotherapy administration.

## Figures and Tables

**Table 1:** Agreement between assessors by symptom grade.

Grade	Assessments *n* (%)	Agreement *n* (%)
**Grade 0**	44 (36)	41 (93)
**Grade 1**	29 (24)	26 (90)
**Grade 2**	33 (27)	26 (79)
**Grade 3**	13 (11)	13 (100)
**Grade 4**	3 (2)	3 (100)

**Table 2:** Time taken for assessor to complete chemotherapy induced phlebitis severity (CIPS) scale (V8).

Grade	Number of Assessments	Mean Time Taken
0	9	1 min 8 s
1	8	1 min 42 s
2	7	2 min 10 s
3	1	1 min 14 s
4	0	NA
All grades	25	1 min 36 s

**Table 3:** Patient reported symptom severity compared with CIPS scale.

CIPS Scale Grade	Patient Reported Symptom Severity			
	None *n* = 66	Mild *n* = 101	Moderate *n* = 55	Severe *n* = 7
Grade 0	38 (58%)	3 (3%)	0	0
Grade 1	19 (29%)	59 (58%)	3 (5%)	0
Grade 2	5 (8%)	33 (33%)	17 (31%)	0
Grade 3	4 (6%)	5 (5%)	30 (55%)	5 (71%)
Grade 4	0	1 (1%)	5 (9%)	2 (29%)

The comparison of patient reported severity of symptoms with staff assessment of grade using the CIPS scale is illustrated with red indicating the highest proportion of CIPS grade recorded for each level of participant reported symptoms, amber illustrating where the proportion was <10% and green where it was zero.
